# A GIS-Based Artificial Neural Network Model for Spatial Distribution of Tuberculosis across the Continental United States

**DOI:** 10.3390/ijerph16010157

**Published:** 2019-01-08

**Authors:** Abolfazl Mollalo, Liang Mao, Parisa Rashidi, Gregory E. Glass

**Affiliations:** 1 Department of Geography, University of Florida, 3141 Turlington Hall, P.O. Box 117315, Gainesville, FL 32611, USA; liangmao@ufl.edu (L.M.); gglass@epi.ufl.edu (G.E.G.); 2 Department of Biomedical Engineering, University of Florida, 1064 Center Drive, NEB 459, Gainesville, FL 32611, USA; parisa.rashidi@bme.ufl.edu; 3 Emerging Pathogens Institute, University of Florida, Gainesville, FL 32611, USA

**Keywords:** Artificial neural networks, geographic information system, hotspot detection, multilayer perceptron, Tuberculosis

## Abstract

Despite the usefulness of artificial neural networks (ANNs) in the study of various complex problems, ANNs have not been applied for modeling the geographic distribution of tuberculosis (TB) in the US. Likewise, ecological level researches on TB incidence rate at the national level are inadequate for epidemiologic inferences. We collected 278 exploratory variables including environmental and a broad range of socio-economic features for modeling the disease across the continental US. The spatial pattern of the disease distribution was statistically evaluated using the global Moran’s *I*, Getis–Ord General *G*, and local Gi* statistics. Next, we investigated the applicability of multilayer perceptron (MLP) ANN for predicting the disease incidence. To avoid overfitting, L1 regularization was used before developing the models. Predictive performance of the MLP was compared with linear regression for test dataset using root mean square error, mean absolute error, and correlations between model output and ground truth. Results of clustering analysis showed that there is a significant spatial clustering of smoothed TB incidence rate (*p* < 0.05) and the hotspots were mainly located in the southern and southeastern parts of the country. Among the developed models, single hidden layer MLP had the best test accuracy. Sensitivity analysis of the MLP model showed that immigrant population (proportion), underserved segments of the population, and minimum temperature were among the factors with the strongest contributions. The findings of this study can provide useful insight to health authorities on prioritizing resource allocation to risk-prone areas.

## 1. Introduction

Tuberculosis (TB) is a contagious disease caused by *Mycobacterium tuberculosis* [1]. The disease is primarily transmitted through the respiratory route by coughing or sneezing [2]. The disease mostly attacks the lungs but can also affect other organs such as kidney and brain [3]. It can promote the course of human immunodeficiency virus (HIV) infection into acquired immune deficiency syndrome (AIDS) [4]. According to the World Health Organization (WHO) global TB report, it is estimated that 10.4 million incident cases in 2016 developed the disease, of which almost 1.7 million patients died [5]. This agency has ranked TB as the leading cause of death among HIV patients, the most common killer from a single infectious agent, and the 9th leading cause of death, worldwide.

According to the statistics by the WHO, most TB cases (>90%) are reported in developing countries; however, it can also occur in developed countries [6,7]. Despite efforts to eradicate TB in the US, the disease remains a major public health challenge [7]. In 2016, more than 9200 TB cases were reported in various parts of the US, which placed the disease among the top notifiable infectious diseases in the country [8]. Although the frequency of TB has decreased in recent years, it is not expected that the US will achieve the goal of TB elimination in this century [9].

There are many factors that influence the spatial distribution of TB, which has made the disease a multidimensional and complex public health problem [10,11,12,13]. Previous researches from different parts of the world have demonstrated that TB transmission is related with various individual factors, for example, age, gender, education level, race, migration, drinking alcohol, and presence of diseases (such as HIV and diabetes) [14,15,16]. Moreover, at the ecological level, factors such as climate, altitude, air pollution, economic level, unemployment rate, and poverty have found significant on TB occurrence [17,18]. One of the major drawbacks of the highly applied traditional statistical models in the study of TB is that these models are often based on several hard-to-meet assumptions [19,20]. This can bias the estimations of TB frequency/ incidence rate [21]. For instance, some assumptions of the linear regression (LR) model are normality of all variables, the linear relationship between inputs and output, constant variance of errors, and little or no multicollinearity. They also often need a complete and/or long-term recorded dataset to achieve unbiased estimations [22]. On the other hand, machine learning techniques (MLTs) may lead to appropriate estimations even with noise-contaminated and incomplete data [23,24]. As advanced tools, MLTs have been successfully used in analyzing and modeling various complex environmental disciplines, including in ecology, geography, biomedicine, and epidemiology [25,26,27,28,29]. The growing popularity of MLTs can be attributed to their abilities to approximate almost any complex non-linear functional relationship [30,31,32]. Despite their capabilities in working with noisy and incomplete data as in most epidemiological studies, they have been underused in spatial epidemiology [33,34].

Inspired by human neural processing, artificial neural networks (ANNs) are among the most popular MLTs used in recent years in environmental studies. Artificial neural networks have a large number of highly interconnected processing elements (neurons) working in unison to solve specific problems [35]. Compared to the traditional statistical models, ANNs are independent of the statistical distribution of data and do not require a priori knowledge about the data for deriving patterns. ANNs are simplified mathematical models that can map the relationship between input and output layers by receiving several examples (training data). A properly trained network can further be used to predict outcome(s) from new data (test data) [25].

There have been few published ANN architectures in spatial modeling of infectious diseases, worldwide. Aburas et al. applied an ANN model with a back-propagation algorithm to predict the frequency of confirmed dengue cases using Singaporean National Environment Agency data [36]. Results of their model showed a correlation coefficient of 0.91 between actual and predicted values. They also identified influential environmental factors predicting the number of dengue cases including mean temperature, mean relative humidity, and total rainfall. Laureano-Rosario et al. used ANN to predict dengue fever (DF) occurrence in a region in Puerto Rico, and in several coastal municipalities in Mexico [37]. The developed ANN models were trained with 19 years of DF data for Puerto Rico and six years’ data for Mexico. Sea surface temperature, precipitation, air temperature, humidity, previous DF cases, and population size were used as explanatory variables. Their results showed that the ANN successfully modeled DF outbreak occurrences with the overall power of 70% in both areas. The variables with the most influence on predicting DF outbreak were population size, previous DF cases, air temperature, and date. In the US, Xue et al. developed the least square (LS) regression analysis and a neural network trained by the genetic algorithm to evaluate influenza activity in 10 geographic regions [38]. They compared the models using three evaluation metrics: mean square error, mean absolute percentage error, and relative mean square error. All three evaluation indices used in their study were lower than the corresponding metrics for the LS regression model showing the superiority of the genetic algorithm-based neural network to the LS regression model. 

To date, TB control efforts have relied on the empirically developed WHO DOTS (directly observed treatment, short-course) control strategy which focuses on “case-finding” rather than “place-finding” [39]. Previous studies from different parts of the US and at different levels have shown the association of TB with socio-economic status. Mullins et al. used purely spatial scan statistics to identify spatial clusters of census tracts with high TB prevalence rates in Connecticut. They found six clusters of TB containing 126 census tracts [40]. Persons in these clusters were more likely to be black non-Hispanic and less likely to be Asian. Bennett et al. used multivariate logistic regression to assess the association between demographic and clinical characteristics and latent TB infection in refugees in San Diego, California [41]. They found that the highest prevalence rate was among refugees from sub-Saharan Africa and those with less education. In a study in Harris County (in Texas), Feske et al. showed a positive association between the percent of individuals using public transportation in census tract and location of clusters detected by Getis–Ord’s Gi* hotspot analysis [42].

Ecological level researches on TB incidence rate at the national level are inadequate for epidemiologic inferences especially in the US. Therefore, it is crucial to perform an ecological study across the continental US to identify the location of statistically significant hotspots and to determine the relationship between environmental and socio-economic factors and TB incidence to provide useful insight to policymakers in planning for TB control at a larger scale. To our knowledge, no study has utilized ANNs in modeling the geographic distribution of TB incidence rate in the US. Integration of the GIS and ANN can improve policymakers’ insight in identifying potential TB high-risk areas and risk factors useful for future mitigation efforts. We examined the spatial distribution of the disease and applicability of MLTs in TB modeling with the following assumptions (1) all reported county-level TB incidence rates represent the status of TB in the continental US and (2) the TB incidence rate is influenced by environmental and socioeconomic factors.

## 2. Material and Methods

### 2.1. Tuberculosis Data

Data on all reported TB cases in the continental US between 2006 and 2010 were obtained from the paper of Scales et al. in the American Journal of Preventive Medicine [43]. All data are at the county level (*n* = 3109) and are publicly available [44]. Latent TB cases were not reported and included in this study. To alleviate variations of TB incidences, particularly in counties with a small population size such as Loving and King counties in Texas (*n* < 300 populations), the cumulative incidence was calculated (2006–2010). For this purpose, five-year corresponding population estimates from the American Community Survey (ASC) [45] were used. Tuberculosis incidence rates were imported into ArcGIS 10.5 (ESRI, Redlands, CA, USA) and geocoded at the county level.

### 2.2. Explanatory Data

In this study, 278 environmental and socio-economic factors were collected from various sources and considered as explanatory variables based on previous studies and domain knowledge. From the Center for Disease Control and Prevention (CDC) wonder database [46], climate data were derived including daily maximum and minimum air temperature (°F), and daily maximum heat index (°F). Also, the average number of diabetes cases [47] during the study period were obtained from this database. Topographic data including minimum, maximum and mean of altitude and slope were obtained from the national map website [48]. The county-level values of these data were calculated using zonal statistics in ArcGIS Spatial Analyst extension. Socioeconomic data were acquired from the US Census Bureau [49]. A broad range of socio-economic factors including age group, agriculture, immigration, education level, employment rate, health, Hispanic or Latino population, income, poverty, and race were obtained across the nation from this database. All population-based predictors were normalized to the county’s population size. The full dataset description of exploratory variables is in Supplementary Table S1. All data used in this study are downloadable from the above sources.

### 2.3. Global and Local Clustering

After mapping TB incidence rates at the county level, the empirical Bayes smoothing method was implemented in the GeoDa software [50] to adjust the crude incidence rates toward the global mean. This helps to reduce the variance instability associated with counties with a small population size [51]. Thus, the response variable changed to (logged) smoothed TB incidence rate (STIR) rather than the crude incidence rate with more than 900 counties with 0 value which makes modeling difficult. All these counties have non-zero STIR values. Next, the spatial pattern was statistically evaluated using the global Moran’s *I* and Getis–Ord General *G*. 

Global Moran’s *I* measures the similarities between the TB incidence rates of neighboring counties as follows [52,53]:

(1) $$I=\frac{n\sum_{i=1}^{n}\sum_{j=1,j\neq i}^{n}w_{ij}z_{i}z_{j}}{s_{0}\;\sum_{i=1}^{n}z_{i}^{2}}$$

(2) $$s_{0}=\sum_{i=1}^{n}\sum_{j=1}^{n}w_{ij}$$

where $z_{i}$ and $z_{j}$ are the deviations of STIR for counties i and j from average incidences (i.e., $\left(x_{i}-\bar{x}\right),\;\left(x_{j}-\bar{x}\right)$), respectively; $w_{ij}$ is the spatial weight based on Rook’s contiguity (i.e., common borders between counties i and j); and $s_{0}$ is the aggregation of all spatial weights.

Moreover, the Getis–Ord General *G* statistics, developed by Getis and Ord was used as a measure of clustering of the high or low value of STIR [54]. A positive or negative Z-score for G indicates spatial clustering of high (hotspot) or low (coldspot) values, respectively. The formula for the general *G* of spatial association is:

(3) $$G=\frac{\sum_{i=1}^{n}\sum_{j=1}^{n}w_{ij}x_{i}x_{j}}{\sum_{i=1}^{n}\sum_{j=1}^{n}w_{ij}}\forall j\neq i$$

Getis–Ord Gi* statistics [55] was applied on the smoothed rates to identify the locations of statistically significant hotspots of the STIR (*p* < 0.05). Using the same notation as in Equations (1) and (2), this statistic is computed as follows [42,51]:

(4) $$G_{i}^{*}=\frac{\sum_{j=1}^{n}w_{ij}x_{j}-\bar{X}\sum_{j=1}^{n}w_{ij}}{S\;\sqrt{\frac{\left[n\sum_{j=1}^{n}w_{ij}^{2}-{\left(\sum_{j=1}^{n}w_{ij}\right)}^{2}\right]}{n-1}}}$$

(5) $$S=\sqrt{\frac{\sum_{j=1,j\neq i}^{n}{\left(x_{j}-\bar{x}\right)}^{2}}{n-1}-{\bar{x}}^{2}}$$

### 2.4. Artificial Neural Networks

**Figure 1:**
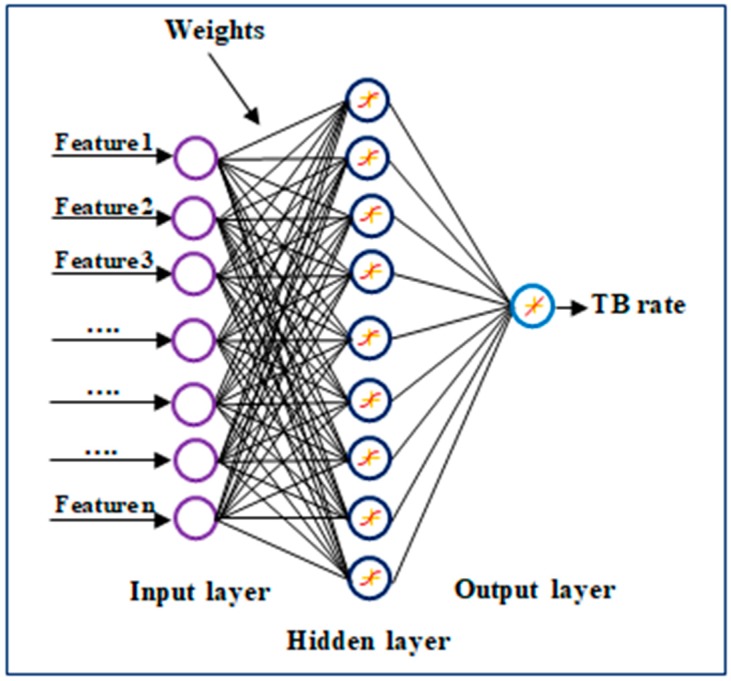
Topological architecture of multi-layer perceptron neural network (MLPNN) used in this study [63].

An ANN is a computational model, which consists of several simple processing elements called neurons [56]. The neurons are usually structured in layers: the input layer, the hidden layer(s) and the output layer. In this study, we used ANNs with one and two hidden layers, however, theoretical research has shown that almost any complex and non-linear function can be estimated by an ANN with a single hidden layer [57]. Additionally, having more hidden layers increases the number of parameters, which may lead to over-fitting (i.e., memorizing the data while training). The aim of the hidden layer is to find a multi-dimensional expansion of the input layer, which can be better transformed to the pattern in the output layer [58]. The neurons in the input layer are connected to all neurons in the hidden layer. Similarly, all the neurons in the hidden layer are connected to every neuron in the output layer (Figure 1). In this system, selected explanatory variables are fed to the input layer and passed through the hidden layer that processes them using simple mathematical operations. The relationship between input and output layers of ANNs is trained by observing a series of known examples from the training dataset and adjusting the weights accordingly. Once the training phase is completed, the network is usually able to generalize what it has learned to the test data with similar attributes of input. We developed a multi-layer perceptron (MLP) neural network with one and two hidden layers to approximate the dependency of log (STIR) in the continental US to environmental and socio-economic factors.

Multi-layer perceptron is the most commonly used ANN structure in environmental modeling [59,60]. During the training phase of MLP, each input feature is multiplied by its corresponding weight. The results are then summed and passed through a smooth non-linear activation function to produce the output. The Logistic function and hyperbolic tangent are among the widely used activation functions [61,62]. In a supervised learning setup, the difference between the (MLP) output and actual output/target (i.e., the error or cost function) can be calculated as in Equation (5) [63]:

(6) $$error=\frac{1}{2}\sum_{i=1}^{n}{\left(t_{i}-o_{i}\right)}^{2}$$

where o and t are model output and target respectively, and n is the training sample size. To minimize the cost function, a back-propagation algorithm based on stochastic gradient descent is used to adjust each weight in the MLP model. The updated value for each weight is calculated as in Equation (6):

(7) $$w^{\left(k+1\right)}:=w^{\left(k\right)}-\alpha \frac{\partial error}{\partial w^{\left(k\right)}}$$

where α or learning rate controls how much the coefficients can change on the kth update. More detailed information about back-propagation MLP is presented in [64,65].

### 2.5. Model Pre-Processing

**Figure 2:**
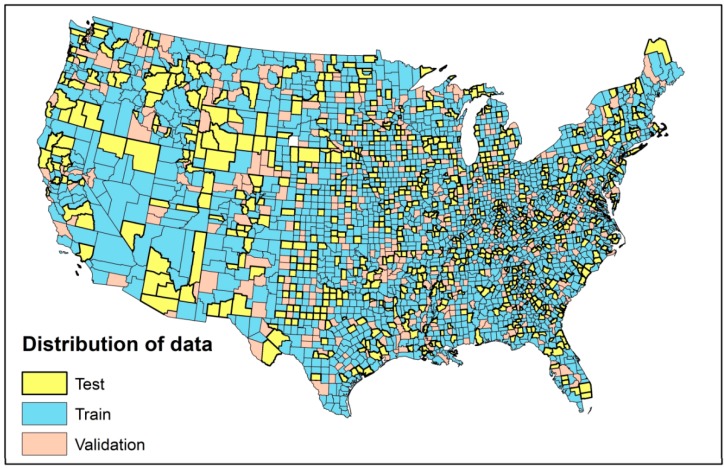
Spatial distribution of training, cross-validation, and test data used for modeling log (STIR).

To develop the models, the entire dataset was randomly divided into three different partitions: (1) training data: to learn and update the weights and biases in network (60% of the total data) (2) cross-validation data: to avoid overfitting problem by tuning models’ parameters during training phase (15% of the total data) (3) test data: to evaluate accuracy and predictive power of the network after the training process (25% of the total data). The spatial distribution of training, cross-validation, and test data as shown in Figure 2. For the purpose of comparison, we used the same training, cross-validation and test data for all developed models.

The next step was to standardize the input data before using them in the ANN models. Input data have different ranges and units, this pre-processing step can enhance the performance of models by faster convergence [25,66]. There are several standardization formulas presented in the literature. Here, we use the following equation which transforms the input data to the range of 0 to 1 as in Equation (8):

(8) $$X_{s}=\frac{X_{i}-X_{min}}{X_{max}-X_{min}}$$

where $X_{i}$ is the initial (actual) value; $X_{min}$ and $X_{max}$ are the minimum and maximum of the initial values and $X_{s}$ is the respective standardized value. Moreover, after training the network, the output of the model is returned to the original form through the Equation (9):

(9) $$X_{i}=X_{s}*\left(X_{max}-X_{min}\right)+X_{min}$$

Multi-layer perceptron and linear regression (LR) models are sensitive to redundant explanatory variables because noise reduces model accuracy and generalizability. Thus, a ‘proper’ selection of independent variables is crucial for better performance. We applied L1-regularization or least absolute shrinkage and selection operator (LASSO) on the training and cross-validation dataset before developing the models. This process produces a sparse solution (i.e., few non-zero coefficients) which reduces overfitting and enhances interpretability of the results [67]. Detailed information of L1 regularization technique can be found in Park and Hastie [68].

In the MLP model, 8 and 1 neurons were used in the input and output layers, respectively. These numbers correspond to the 8 explanatory variables selected during L1 regularization and one response variable (i.e., log (STIR)). There is no deterministic rule to determine the number of neurons in the hidden layer. The grid search was used to tune hyper-parameters in MLP with one and two hidden layer(s). This approach systematically evaluates the developed model for each combination of model parameters. For MLP, the tangent hyperbolic activation function $\left(\emptyset \left(x\right)=\frac{1-e^{-x}}{1+e^{-x}}\right)$, a non-linear and symmetric function which maps any real value to [−1, 1], was used in the hidden layer(s) [69]. In addition, a linear identity function (f(x)=x) was applied as an activation function in the output layer. All computer codes were developed in the Python programming language (Supplementary Material).

### 2.6. Model Evaluation

We computed three types of evaluation metrics to assess and compare the generalization capability of MLP with LR in predicting log (STIR) in the continental US. These statistics include root mean square error (RMSE), mean absolute error (MAE) and correlation coefficient (R) between model output and ground-truth.

Due to the fully connected architecture of MLP, it is difficult to define the explicit relationship between input and output variables by coefficients [70]. Sensitivity analysis is a common way to address this problem. In this analysis, each factor was excluded from the model, individually, and the RMSE of resulting models were compared. The most influential factor, among the selected features, is the one that its absence increases the RMSE of the model the most. Using sensitivity analysis, we identified the most influential factors in predictions of the log (STIR). Finally, we ranked them according to their decreasing importance.

## 3. Results

**Figure 3:**
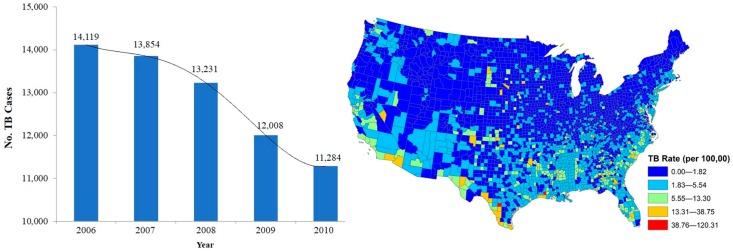
The frequency of TB cases (**left**) and the cumulative TB incidence rate (**right**) across the continental US (2006–2010).

Between 2006 and 2010, 64,496 TB cases were reported across the continental US. The number of TB cases showed a consistent declining trend from 14,119 to 11,284 annual cases (Figure 3). Among the states, the highest average TB incidence rates were identified in Louisiana (5.30 cases per 100,000), Arizona (5.05 per 100,000) and Georgia (4.7 per 100,000).

**Figure 4:**
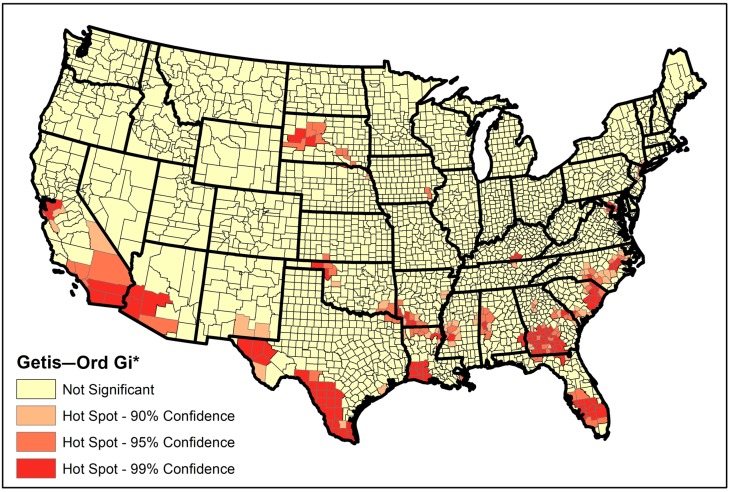
Hotspot map for the STIR in the continental US identified by hotspot analysis (Getis–Ord Gi*) technique, 2006–2010.

The global Moran’s *I* and general *G* indicated significant spatial clustering of STIR in the continental US for the study period (Moran’s *I* = 0.13, Z-score = 32.13, *p* < 0.005; General *G* = 0.002, Z-score = 15.3, *p* < 0.005). The hotspot analysis (Getis–Ord Gi*) identified that about 7% of the continental US counties (*n* = 216) were part of hotspots. The hotspots of STIR were distributed unequally, almost restricted to the southern half of the country and particularly in the southern and southeastern counties of the US (Figure 4). Table 1 summarizes the top 10 states with the largest number of counties detected as part of STIR hotspots by the Getis–Ord Gi* technique.

Based on the results of L1 regularization, the environmental and several socio-economic factors were selected. Out of 278 explanatory variables, only 8 factors were incorporated as input variables for the models: (1) RHI820: resident population: not Hispanic, white alone (July 1-estimate) (proportion of county population); (2) LFE330: employed persons by industry (NAICS)-agriculture, forestry, fishing and hunting, and mining (proportion of county population); (3) minimum temperature; (4) POP778: year of entry by citizenship status in the United States entered 2000 or later-foreign-born (proportion of county population) (5) IPE110: people of all ages in poverty (proportion of county population); (6) SPR440: social security-benefit recipients (proportion of county population); (7) HIS305: Hispanic or Latino persons, educational attainment, 25 years and over, male (proportion of county population); (8) POP730: population one year and over by residence-moved from different county within same state 2005–2009 (proportion of county population).

All Pearson correlation values among the selected variables were under 0.5, thus we considered the selected factors as relatively uncorrelated (Table 2). The log (STIR) was positively correlated with all variables except for household income (*p* < 0.05).

Preliminary results of the developed LR model showed that the selected predictors generated *R* = 0.666, *R^2^* = 0.443, and *F* = 184.246 (Sig. F Change < 0.001). The *R* value which represents the simple correlation between predictions and reality indicated an acceptable degree of correlation. The *R^2^* value indicates that 44.3% of total variations in the log (STIR) can be explained by the predictors. The *F*-test was significant showing the developed LR model as a whole has statistically significant predictive capability. Durbin–Watson test was close to 2 which verifies independency of errors assumption (Durbin–Watson statistic = 2.04) (Table 3).

The *t*-test showed that all selected variables are statistically significant at a 99% confidence interval. Based on the standardized coefficients, among the variables, in order of strength, “RHI820”, “LFE330”, “HIS305”, “SPR440”, and “POP730” have negative impacts on the log (STIR) while the variables “POP778”, “Min Temp”, and “IPE110” have positive impacts on the dependent variable, respectively. Tolerance and the variance inflation factor (VIF) were used as two collinearity diagnostic tests to assess multicollinearity level for all variables. As the values of VIF didn’t exceed 3 and tolerance statistic were above 0.1, it seems that there is no cause for concern about collinearity (Table 4). 

**Figure 5:**
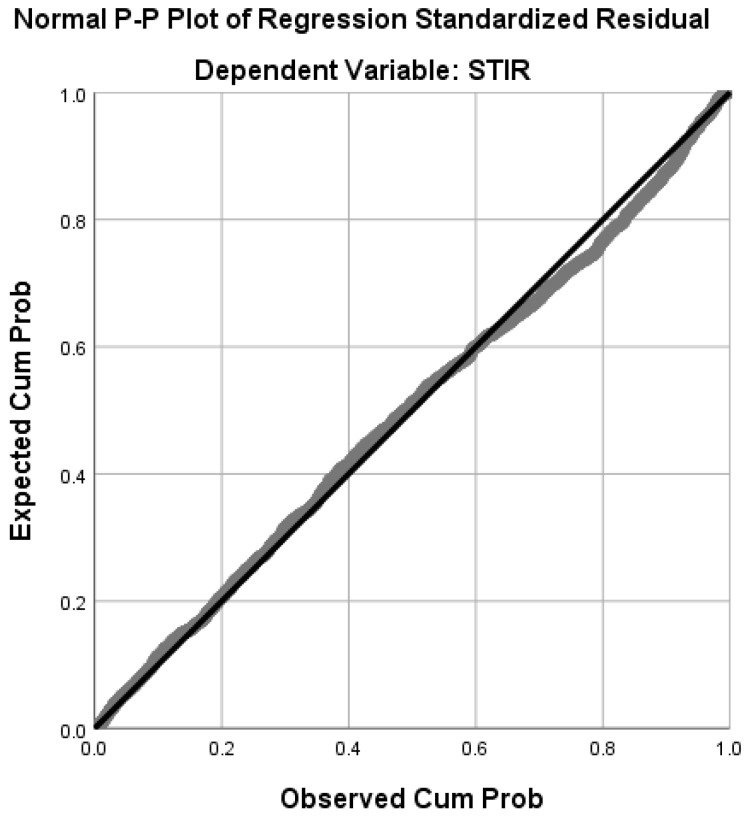
The Normal P-P Plot of LR model.

The normal probability-probability plot (P-P Plot) of LR residuals showed that data points were closely aligned with the diagonal line suggesting the distribution of the residuals was almost normal. This indicated that the assumption of normality of errors was almost met. Nevertheless, there were a few samples which departed from the diagonal line (Figure 5).

Table 5 presents the performance of MLP (1-hidden layer), MLP (2-hidden layer), and LR models, in terms of MAE, RMSE, and R, for training, cross-validation and test datasets, respectively. The correlation coefficient for a single hidden layer MLP, with 20 nodes in the hidden layer, was larger than double hidden layers MLP, and LR (Table 5), which showed a better agreement between the predicted and the ground-truth. In addition, RMSE in MLP (single layer) was lower than the other models. However, MAE in MLP with a two-hidden layer (20 nodes in first and 10 nodes in second hidden layers) had the same test errors as single layer MLP. Results suggest that the single layer MLP model outperformed the rest of models with higher generalizability for predicting the log (STIR) in the continental US. Similarly, double hidden layer MLP outperformed the LR model.

**Figure 6:**
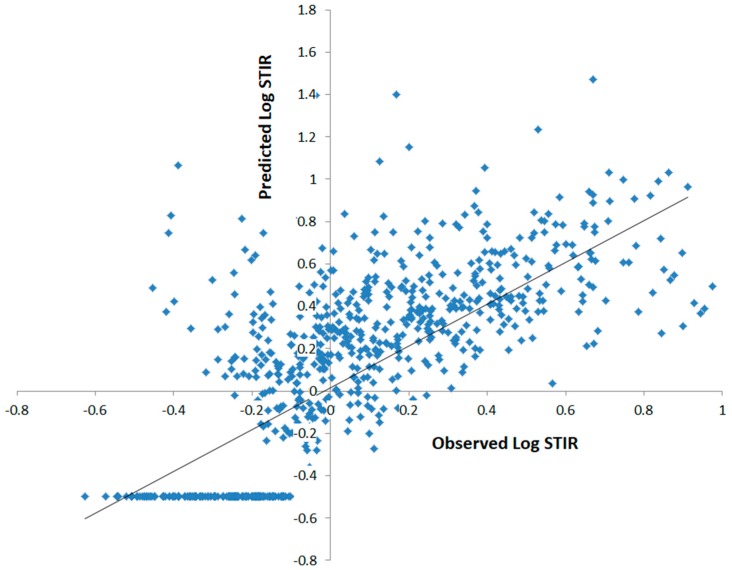
Scatter plot of observed and predicted log (STIR) (by single hidden layer MLP model) for test data in the continental US.

The scatter plot between the output of the MLP model and the corresponding observed log (STIR) (i.e., ground-truth values) for test data (Figure 6) showed that the model was able to predict the average variations of log (STIR), while it was unable to predict some counties with exceptional rates.

**Figure 7:**
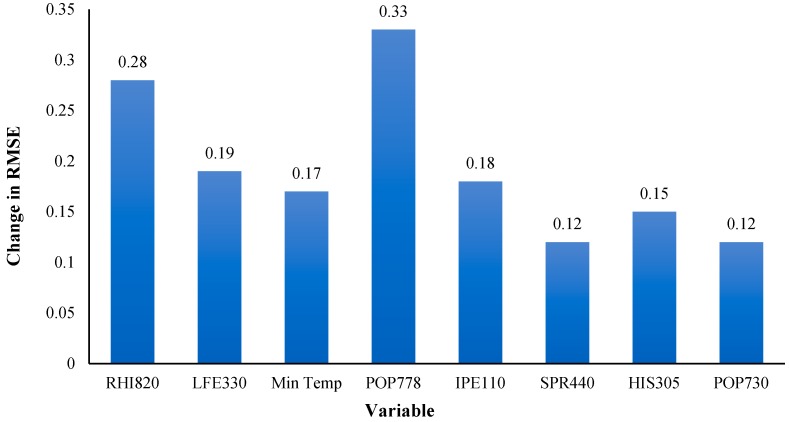
The contribution of input features on predicting log (STIR) according to sensitivity analysis of single hidden layer MLP. RMSE: Root mean square error.

The best model accuracy was achieved by the single layer MLP compared with the other models. We examined the contribution/relative importance of each input feature on the log (STIR) using sensitivity analysis. The results revealed that the highest RMSE occurred when “resident population of American Indian and Alaska native” was removed from the model, which implies that this factor has the maximum contribution, among the selected factors, in predicting log (STIR) at the county level in the continental US. The most influential factors in order of contributions were: “RHI820: resident population: not Hispanic, White alone (July 1-estimate) (proportion)”, “LFE330: employed persons by industry (NAICS)-agriculture, forestry, fishing and hunting, and mining (proportion)”, “Minimum Temperature”, “POP778: year of entry by citizenship status in the United States entered 2000 or later-foreign-born (proportion)”, “IPE110: people of all ages in poverty (proportion)”, “SPR440: supplemental security income-average monthly payments per recipient”, “HIS305: Hispanic or Latino persons, educational attainment, 25 years and over, male (proportion)” and “POP730: population one year and over by residence-moved from different county within same state 2005-2009 (proportion)”. (Figure 7).

## 4. Discussion

According to the Institute of Medicine (2000), eliminating TB will require the development of new tools to identify risk factors and high-risk areas [71]. As expressed by Feske et al., effective TB elimination in the US would require geographic elucidation of high-risk areas and systematic surveillance of location-based risk factors [42]. In this study, we combined more advanced tools to examine the relationship between environmental and socio-economic factors, and the log (STIR) across the continental US. Integration of GIS, spatial statistics, and ANNs resulted in an efficient multi-disciplinary approach, which can provide helpful guidelines for health decision makers. The benefits obtained from this approach can enhance mitigation efforts such as budget allocation, educating people who live in high-risk areas and drug distribution. Due to limited research on the spatial modeling of TB at the national level, our study can be regarded as a basis for future nationwide TB program researches.

In this study, we examined the spatial pattern and hotspots of the STIR in the continental US. Moran’s *I* and General G statistic were used to investigate the presence of spatial autocorrelation of local STIR. Our results showed that the distribution of STIR at the county levels is clustered at the county level (*p* < 0.05). We then conducted the hotspot analysis to identify the counties with statistically significant STIR using the hotspot analysis (Getis–Ord Gi*) approach. Our findings showed that STIR hotspots were concentrated in the southern and western states (Figure 4); however, the South, Southeast, and Southwest counties of the country were more severely affected. The concentration of hotspots suggests that there were more cases observed in these areas that would be expected if everyone were equally at risk. Visual comparison of the location of identified hotspots in some states which had a very high proportion of counties falling into hotspots (such as Georgia and Florida) with recent surveillance reports of Georgia [72] and Florida [73] showed pieces of evidence of similarities with some differences. This suggests the stability of location of counties falling into hotspots which require close attention. Since after detecting the statistically significant hotspots of STIR in the region, associated risk factors were not known, we used ANN to model the relationship between environmental and socio-economic factors and the log (STIR).

Based on sensitivity analysis, the environmental factors identified the contribution of the selected variables to the log (STIR) at the county level. We found that the average daily minimum temperature (with a positive effect) was an important climate factor in the log (STIR). This is probably due to the adverse effects of minimum air temperature on the respiratory system of patients, and more close lifestyle of people in cold weather which increases the risk of exposure to infectious agents [74]. Similar results were reported in other studies. In a time-series analysis study in Fukuoka (Japan), Onozuka and Hagihara found a positive significant relationship between extreme cold temperature and TB incident cases [74]. Our results are also consistent with the findings of Mourtzoukou et al. [75] and Khalid et al. in Pakistan [76].

Sensitivity analysis showed that economic factors are important for log (STIR) in the continental US. One of the most important economic factors was “proportion of population county of all ages in poverty” which suggests that underserved segments of the population are at higher-risk of STIR in the US. Conversely, counties with a higher proportion of the population employed by industry or higher security income had lower STIR. These factors potentially describe the impact of poverty/deprivation on lifestyle choice. These findings agree with the individual-level studies of McKenna et al. [77], Ho [78], and Weis et al. [79]. Unemployment rate has been found to be an important factor in TB transmission in the studies of Munch et al. who indicated the strongest correlation with TB caseload [80], and the studies of Dos Santos [81] and Jackubowiak [82]; while Sun et al. [10] did not find it significant in China. In a cohort study in Georgia, Djibuti et al. [83] showed that TB patients with lower-income households are at higher risk of poor TB treatment. Bamrah et al. analyzed the genotyping of homeless persons in the US, 1994–2010. Their results showed that homeless TB patients had an approximately 10-fold increase in TB incidence and had more than twice the odds of not completing treatments [84].

Results of this study also showed the importance of race distribution and STIR as there was a strong negative relationship between the proportion of county population who are white and STIR. Visual comparison of the locations of the hotspots (Figure 4) with the distribution map of race and ethnicity provided by US Census Bureau [85], confirms that, in general, counties with more than 80% of the white population didn’t fall into the hotspots. This agrees with the findings of Cantwell et al. [11] and CDC report [86].

Artificial neural networks and GIS were effective in modeling log (STIR), but several limitations exist. First, the data used in this study were collected from multiple online sources. It should be noted that TB reported data are subject to spatial differences in case detection, thus, a standard passive case-finding approach needs to be considered. In addition, methods of data collection and preparation are different which may result in biased estimations. In this study, we only included active reported TB cases. It should be noted that there are many people with latent TB infection who have not developed TB disease, and therefore, are not detected or counted as cases. This is more prevalent (exists in greater proportions) among those from other countries. The last limitation stems from the study design. Our study at the county level should be considered to characterize population rather than individual-level characteristics of risk (ecological fallacy). Thus, the findings of this study should be applied to population-level targeting rather than considering individual treatment.

## 5. Conclusions

This study showed machine learning techniques in spatial modeling can be applied to TB incidence rate across the continental US. For future works, we recommend conducting researches at multiple scales, particularly at finer scales such as at the census tracts or block group level for more focal interventions. However, as most policy decisions on TB control are performed at the state and federal government levels, this represents a meaningful first step. To optimize the structure of ANNs, choice of parameters (e.g., learning rate, activation functions) and training the weights in ANN, and heuristic algorithms such as genetic algorithm may be useful leading to global optima, because it can help to escape from local optima [87]. Also, hyper-parameter tuning coupled with more recent ANN approaches in terms of activation function or optimization (e.g., Adam or other adaptive methods) is highly recommended. We also recommend incorporating genetics data into the model or examine the genetic characteristics of individual patients in counties with high modeling errors. In this regard, national TB genotyping surveillance coverage which refers to the proportion of TB cases with culture-positive with at least one genotyped isolate in the US can be useful. Also, other statistical techniques such as Poisson model (through including the size of local population at risk as an offset in the model) or the logistic regression model (through binomial distribution) can be investigated in modeling TB incidence rate. Coupled with GIS, the ANNs techniques successfully identified some determinant factors of log (STIR), ranked them, and had better prediction ability than the traditional linear regression analysis. The findings of this study can provide useful insight to health authorities on prioritizing resource allocation to risk-prone areas.

## Figures and Tables

**Table 1:** Top 10 states with the largest number of hotspot counties (*p* < 0.10) of smoothed tuberculosis (TB) incidence rate (STIR) in the continental US, 2006–2010.

Rank	State	No. Hotspot Counties	Percentage (#hotspots/#counties)
1	Georgia	57	35.8%
2	Texas	30	11.8%
3	North Carolina	23	23.0%
4	Louisiana	22	34.3%
5	Florida	20	29.9%
6	California	17	22.7%
7	South Carolina	17	37%
8	Arkansas	12	16.0%
9	Mississippi	12	14.6%
10	Alabama	10	14.9%

**Table 2:** Pearson correlation analysis between selected variables for modeling STIR, continental US.

	POP730	LFE330	IPE110	POP778	Min Temp	SPR440	HIS305	RHI820
**POP730**	1.000	0.051	0.041	0.064	−0.124	−0.078	−0.138	−0.024
**LFE330**	0.051	1.000	0.018	0.057	0.136	−0.499	−0.186	−0.040
**IPE110**	0.041	0.018	1.000	0.266	−0.231	−0.108	0.066	0.384
**POP778**	0.064	0.057	0.266	1.000	−0.005	0.091	−0.390	0.248
**Min Temp**	−0.124	0.136	−0.231	−0.005	1.000	0.066	−0.032	0.308
**SPR440**	−0.078	−0.499	−0.108	0.091	0.066	1.000	−0.015	0.003
**HIS305**	−0.138	−0.186	0.066	−0.390	−0.032	−0.015	1.000	0.403
**RHI820**	−0.024	−0.040	0.384	0.248	0.308	0.003	0.403	1.000

**Table 3:** Results of linear regression (LR) model for modeling log (STIR), continental US.

	R	R Square	Adjusted R Square	Change Statistics					Durbin–Watson
				R Square Change	F	df1	df2	Sig.	
LR	0.666 ^a^	0.443	0.440	0.443	184.246	8	1854	0.000	2.041

a. Predictors: (Constant), POP73, LFE330, IPE110, POP778, Min Temp, SPR440, HIS305, RHI820. Dependent Variable: log (STIR).

**Table 4:** Effects of environment and socio-economic factors on the log (STIR) using LR model.

	Unstandardized Coefficients	Standardized Coefficients	*t*	Sig.	95.0% Confidence Interval for B		Collinearity Statistics	
**Variables**	**B**	Beta			Lower Bound	Upper Bound	Tolerance	VIF
**(Constant)**	0.001		0.009	0.993	−0.198	0.200		
**RHI820**	−0.007	−0.294	−11.117	0.000	−0.009	−0.006	0.429	2.328
**LFE330**	−0.023	−0.166	−7.929	0.000	−0.029	−0.017	0.683	1.463
**Min Temp**	0.013	0.210	9.809	0.000	0.010	0.016	0.653	1.532
**POP778**	0.083	0.282	12.621	0.000	0.070	0.095	0.602	1.661
**IPE110**	0.012	0.140	6.662	0.000	0.008	0.015	0.677	1.477
**SPR440**	−0.009	−0.097	−4.703	0.000	−0.013	−0.005	0.701	1.426
**HIS305**	−0.019	−0.145	−5.976	0.000	−0.026	−0.013	0.508	1.968
**POP730**	−0.015	−0.080	−4.489	0.000	−0.021	−0.008	0.950	1.053

VIF: Variance inflation Factor.

**Table 5:** Comparison of multi-layer perceptron (MLP; one and two hidden layers), and LR model’ performance for predicting log (STIR) in the continental US.

Model	Training			Cross-Validation			Test		
	MAE	RMSE	R	MAE	RMSE	R	MAE	RMSE	R
LR	0.27	0.35	0.66	0.27	0.36	0.65	0.28	0.36	0.61
MLP (1 hidden layer)	0.25	0.33	0.70	0.26	0.35	0.67	0.27	0.35	0.63
MLP (2 hidden layers)	0.26	0.34	0.69	0.26	0.35	0.65	0.27	0.36	0.62

## Supplementary Materials

The following are available online at http://www.mdpi.com/1660-4601/16/1/157/s1.
